# Comparison of extraction methods for intracellular metabolomics of human tissues

**DOI:** 10.3389/fmolb.2022.932261

**Published:** 2022-08-26

**Authors:** Carolin Andresen, Tobias Boch, Hagen M. Gegner, Nils Mechtel, Andreas Narr, Emrullah Birgin, Erik Rasbach, Nuh Rahbari, Andreas Trumpp, Gernot Poschet, Daniel Hübschmann

**Affiliations:** ^1^ Heidelberg Institute for Stem Cell Technology and Experimental Medicine (HI-STEM gGmbH), Heidelberg, Germany; ^2^ Division of Stem Cells and Cancer, German Cancer Research Center and DKFZ-ZMBH Alliance, Heidelberg, Germany; ^3^ Faculty of Biosciences, Heidelberg University, Heidelberg, Germany; ^4^ Division of Personalized Medical Oncology, German Cancer Research Center, Heidelberg, Germany; ^5^ Department of Personalized Oncology, University Hospital Mannheim, University of Heidelberg, Mannheim, Germany; ^6^ DKFZ-Hector Cancer Institute at the University Medical Center Mannheim, Mannheim, Germany; ^7^ Centre for Organismal Studies (COS), Heidelberg University, Heidelberg, Germany; ^8^ Department of Surgery, Medical Faculty Mannheim, Universitätsmedizin Mannheim, Heidelberg University, Mannheim, Germany; ^9^ German Cancer Consortium (DKTK), Heidelberg, Germany; ^10^ Computational Oncology, Molecular Diagnostics Program, National Center for Tumor Diseases (NCT) Heidelberg and German Cancer Research Center (DKFZ), Heidelberg, Germany

**Keywords:** metabolism, metabolomics, intra-cellular, extraction protocol, absolute quantification

## Abstract

Analyses of metabolic compounds inside cells or tissues provide high information content since they represent the endpoint of biological information flow and are a snapshot of the integration of many regulatory processes. However, quantification of the abundance of metabolites requires their careful extraction. We present a comprehensive study comparing ten extraction protocols in four human sample types (liver tissue, bone marrow, HL60, and HEK cells) aiming to detect and quantify up to 630 metabolites of different chemical classes. We show that the extraction efficiency and repeatability are highly variable across protocols, tissues, and chemical classes of metabolites. We used different quality metrics including the limit of detection and variability between replicates as well as the sum of concentrations as a global estimate of analytical repeatability of the extraction. The coverage of extracted metabolites depends on the used solvents, which has implications for the design of measurements of different sample types and metabolic compounds of interest. The benchmark dataset can be explored in an easy-to-use, interactive, and flexible online resource (R/shiny app MetaboExtract: http://www.metaboextract.shiny.dkfz.de) for context-specific selection of the optimal extraction method. Furthermore, data processing and conversion functionality underlying the shiny app are accessible as an R package: https://cran.r-project.org/package=MetAlyzer.

## Introduction

Recent developments in high-throughput technologies have enabled precise characterization of biological specimens and insight into health and disease. Representing the biological endpoint of the omics cascade, metabolomics is of particular interest and importance with manifold applications, including investigation of neurometabolic disease (Wickenhagen et al., 2009; Wilson et al., 2017; Peters et al., 2020) and insights into infectious disease (Sitole et al., 2013; Chandler et al., 2016; Aurpibul et al., 2020) and the microbiome (Nagy-Szakal et al., 2017; Zimmermann et al., 2019) or cancer (Mimmi et al., 2011; Banerjee et al., 2017).

Technologies for metabolic measurements, which almost exclusively rely on mass spectrometry coupled to various other techniques, for example, gas or liquid chromatography, can be coarsely grouped into untargeted and targeted approaches. While untargeted analyses of the metabolome are particularly well-suited for exploratory projects and hypothesis generation, they most often do not allow absolute quantification of the different metabolites, and many altered features cannot be identified *via* public database searches. Some technologies from the spectrum of targeted metabolomic analyses allow for absolute quantification. Using combinations of internal standards and calibrants, the LC-MS/MS-based Biocrates MxP^®^ Quant 500 kit is an example of such a technology, measures up to 630 different metabolites from various metabolite classes, and provides absolute quantification for a subset of these metabolites, depending on the respective limits of detection (LOD) and the concentrations in the investigated sample type.

Metabolic measurements, especially for absolute quantification, are well-established for the analysis of body fluids (blood plasma (Burla et al., 2018), cerebrospinal fluid (CSF) (Blennow and Zetterberg, 2018), or urine (Zapf et al., 2015; Harpole et al., 2016)). However, measurement of intracellular metabolites requires more complex protocols for their extraction. The pre-analytical phase requires stringent standard operating procedures (SOPs) to limit any variance that may be introduced. It includes disintegration of the 3D tissue architecture and the cellular context, removal of as many extracellular compounds as possible, and lysis of cells. These steps are followed by extraction protocols which are essential for effective quantification of the intracellular abundance of metabolites with heterogeneous chemical characteristics.

In this work, we compare ten different extraction protocols (among which one had two different resolving volumes) for intracellular metabolomic measurements using the Biocrates MxP^®^ Quant 500 kit in four different human sample types. We investigated different tissues (liver and bone marrow) and cell lines [adherent: HEK (“human embryonic kidney” cell line) and non-adherent: HL60 (“human leukemia 60” cell line)]. We further debut our R package for data processing and conversion as well as our R/Shiny app “MetaboExtract” to thoroughly explore this comprehensive dataset, enabling in-depth visualization and analysis of the measured metabolites for a given extraction protocol and sample type.

## Materials and methods

### Samples

Four different sample types were analyzed within this study representing cell culture and primary sample conditions: non-adherent HL60 cells, adherent HEK cells, primary human liver cells, and primary human total bone marrow cells. All primary human material in this study was obtained and used following institutional review board approval by the Medical Ethics Committee II of the Medical Faculty Mannheim, University of Heidelberg, Heidelberg, Germany in accordance with the Declaration of Helsinki after informed written consent.

### Human bone marrow sample preparation

Bone marrow aspiration from a 39-year-old healthy male volunteer was performed according to standard clinical protocols. Mononuclear cells (MNCs) were isolated from fresh bone marrow (BM) by Ficoll density gradient centrifugation. To do so, BM was diluted 1:2 with phosphate-buffered saline (PBS) and loaded on top of Ficoll without disturbing the layer. Samples were centrifuged at 400 x g at room temperature for 30 min. MNCs were extracted and washed with PBS twice. Finally, 3 × 10^6^ cells per sample were collected and snap-frozen using liquid nitrogen.

### HL-60 and human embryonic kidney sample preparation

HL60 cells were kept under cell culture conditions in DMEM Glutamax with 10% FCS and 1% penicillin/streptomycin. HEK cells were kept under cell culture conditions in DMEM Glutamax with 10% FCS and 1% penicillin/streptomycin. Cells were washed twice with ice-cold PBS, and aliquots of 3 × 10^6^ cells were snap-frozen using liquid nitrogen.

### Human liver sample preparation

The liver sample was obtained during surgical liver resection of a 75-year-old male patient with hepatocellular carcinoma. Immediately after surgical resection, a piece of healthy liver tissue was washed with ice-cold 0.9% NaCl solution and snap-frozen in liquid nitrogen. The tissue was pulverized to a fine powder without defrosting using a ball mill (2 × 30 s; 30 Hz; MM 400; Retsch) and pre-cooled stainless-steel beakers. Until extraction, all human samples were stored at −80°C in 30-mg aliquots.

### Extraction

**FIGURE 1:**
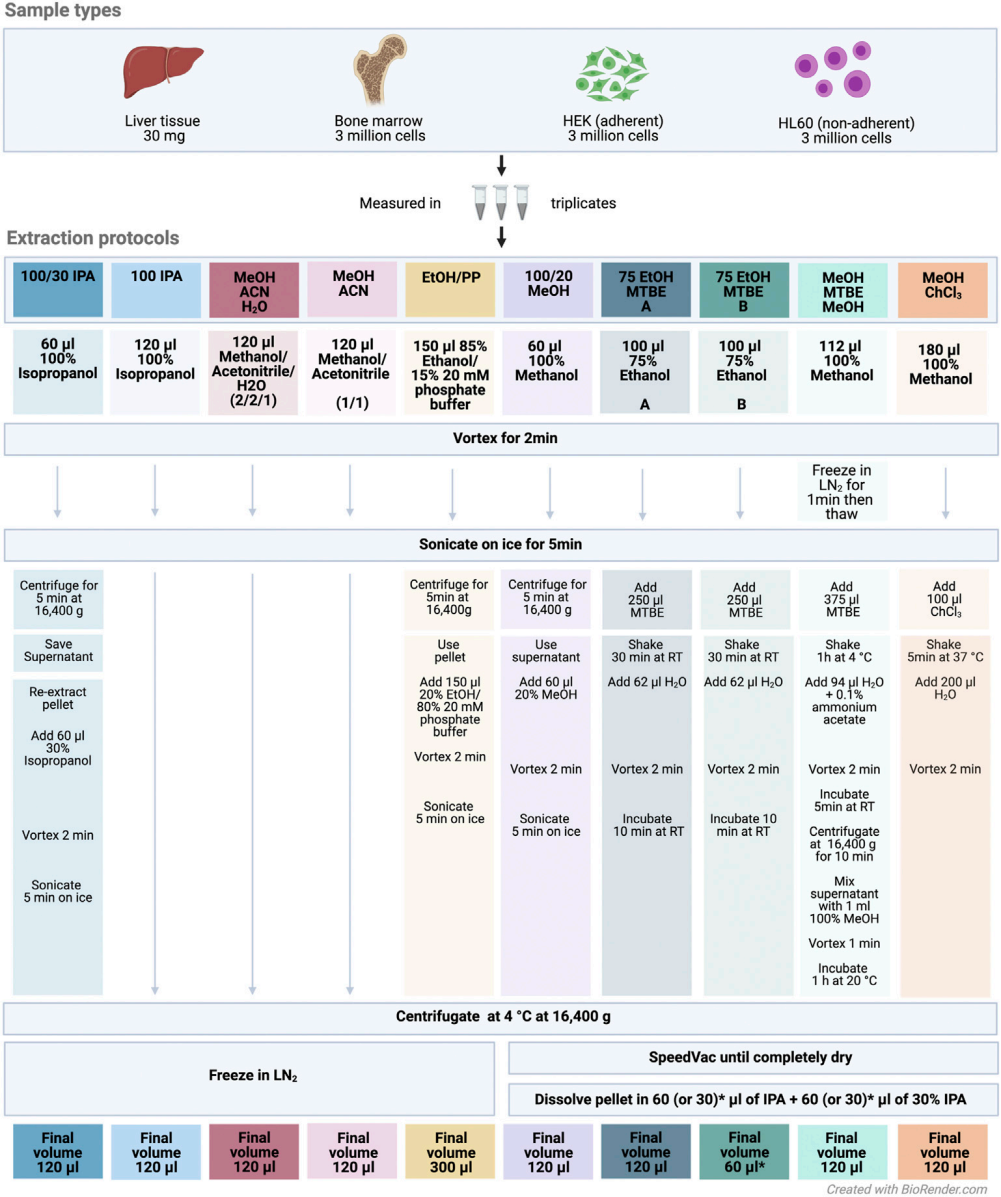
Experimental design and extraction protocol overview. Abbreviations: IPA, isopropanol; MTBE, methyl tert-butyl ether; ACN, acetonitrile; EtOH, ethanol; MeOH, methanol; PP, polypropylene; ChCl3, chloroform; LN2, liquid nitrogen; RT, room temperature. All sample types were measured in triplicates across the extraction protocols. The colors denote the different extraction protocols.

We evaluated ten different extraction protocols as described in Figure 1. We developed or adapted these protocols based on our own preliminary experience and literature review (Lisec et al., 2006; Rabinowitz and Kimball, 2007; Ivanisevic et al., 2013; Weir et al., 2013; Lee et al., 2014; Zukunft et al., 2018) or the Biocrates SOP for processing of cell pellets for analyses *via* AbsoluteIDQ p180.

The protocol 75 EtOH/MTBE was applied with two resolving volumes A) 120 μl and B) 60 μl. Briefly, frozen cell or liver samples were extracted using the indicated solvents and subsequent steps of the respective protocol (Figure 1). An ultrasonication ice-bath Transsonic T460 (Elma) was used in some protocols as depicted. After a final centrifugation step, the solvent extract of the protocols *1: 100/30 IPA*, *2: 100 IPA*, *3: MeOH/ACN/H2O*, *4: MeOH/ACN*, and *5: EtOH/PP* was transferred into a new 1.5-ml tube (Eppendorf) and snap-frozen until kit preparation. For the remaining protocols, the supernatant (in the biphasic extractions with MTBE or chloroform in both phases) was dried using an Eppendorf Concentrator Plus set to no heat and stored at −80°C and reconstituted in 120 or 60 μl (see above) isopropanol (60 or 30 μl of 100% isopropanol, followed by 60 or 30 μl of 30% isopropanol in water) directly before further processing according to the MxP Quant 500 manual.

All chemicals and solvents used were of UHPLC-MS grade quality (Sigma-Aldrich, Germany).

### Sample analysis and analysis

In total, 630 metabolites covering 14 small molecules and nine different lipid classes were analyzed using the MxP^®^ Quant 500 kit (Biocrates) following the manufacturer’s protocol. For a full list of metabolites covered, we refer to the specification of the Biocrates MxP^®^ Quant 500 kit [https://biocrates.com/wp-content/uploads/2021/01/biocrates-Quant500-list-of-metabolites-v4-2021.pdf] (Supplementary Table S1). Of note, 12 different lipid classes are summarized into nine by the MetIDQ software (Biocrates) merging lysophosphatidylcholines and phosphatidylcholines to glycerophospholipids as well as hexosyl-, dihexosyl- and trihexosylceramides to glycosylceramides. In brief, 10 µl of the extract was pipetted on a 96 well-plate containing internal standards and dried under a nitrogen stream using a positive pressure manifold (Waters). Fifty µl of a 5% phenyl isothiocyanate (PITC) solution was added to each well to derivatize amino acids and biogenic amines. After 1 h incubation time at room temperature, the plate was dried again. To extract the metabolites, 300 µl 5 mM ammonium acetate in methanol was pipetted to each filter and incubated for 30 min. The extract was eluted into a new 96-well plate using positive pressure. The pipetting order of samples was randomized before application onto the 96 well plates. For further LC-MS/MS analyses, 150 µl of the extract was diluted with an equal volume of ultra-pure water. For FIA-MS/MS analyses, 10 µl extract was diluted with 490 µl of FIA solvent (provided by Biocrates). After dilution, LC-MS/MS and FIA-MS/MS measurements were performed. For chromatographic separation, a UPLC I-class PLUS (Waters) system was used coupled to a SCIEX QTRAP 6500 + mass spectrometry system in electrospray ionization (ESI) mode. Data were recorded using the Analyst (Sciex) software suite and transferred to the MetIDQ software (version Oxygen-DB110-3005) which was used for further data processing, that is, technical validation, quantification, and data export. Metabolite-specific LODs are listed in Supplementary Table S2. All metabolites were identified using isotopically labeled internal standards and multiple reaction monitoring (MRM) using optimized MS conditions as provided by Biocrates. For quantification, either a seven-point calibration curve or one-point calibration was used depending on the metabolite class. The analytical method has been fully validated by the kit manufacturer according to FDA and EMA guidelines.

We like to mention that FIA-MS/MS analysis does not provide specific information regarding either the positions or chain lengths of the fatty acid residues linked to each lipid’s backbone. As a consequence, the detected signal is a sum of several isobaric/isomeric lipids or acylcarnitines. For a list of isobaric/isomeric lipid species or acylcarnitines, we refer to the detailed corresponding documentation of the vendor [https://biocrates.com/wp-content/uploads/2020/02/Biocrates_Q500_isomers_isobars.pdf]. Examples of UPLC-MS/MS chromatograms of QC, cell, and tissue samples can be found in Supplementary Figures S1–S3.

### Data analysis

Downstream analysis of MetIDQ output was performed using custom R scripts (v4.0.0). The code used for the figures in this study is available at https://github.com/andresenc/extractioncomparison. ggplot2 (v3.3.3) and cowplot (v1.1.1) were used for generation of plots. For data transfer from MetIDQ into R, we implemented the R package MetAlyzer (https://CRAN.R-project.org/package=MetAlyzer), which facilitates the reading of standardized output files, convenient data handling, statistics, and downstream analysis.

Metabolites were defined as “above LOD” when at least two replicates met this criterion. For PCA, raw data were filtered for metabolites that were below LOD in all samples, and zero values were replaced by taking the minimum of all measured concentrations per metabolite and adding 20% to this value, and data were log2-transformed and scaled using the Pareto method as described by van den Berg et al. (2006). To identify associations between principal components and the experimental setting, the Kruskal–Wallis test was applied. *p*-values were corrected using Benjamini–Hochberg and considered significant if p. adjust <0.05.

To compare concentration yields between extraction protocols, an ANOVA was calculated based on log2-transformed data. The ten extraction protocols were used as the categorical variable and concentration as the dependent variable. Using the Tukey post-hoc test group labeling, the optimal extraction methods with the highest median yield were determined for each metabolite and sample type.

## Results

Cells or tissues need to be lysed, and metabolites have to be extracted to analyze intracellular metabolite concentrations. We compared ten different extraction protocols on four sample types and assessed their effect on the abundance and repeatability of the determined metabolite concentrations and on the number of detectable metabolites, that is, yielding concentrations above the limit of detection (LOD) (Figure 1).

### Effective quantification depends on sample type and extraction protocol

**FIGURE 2:**
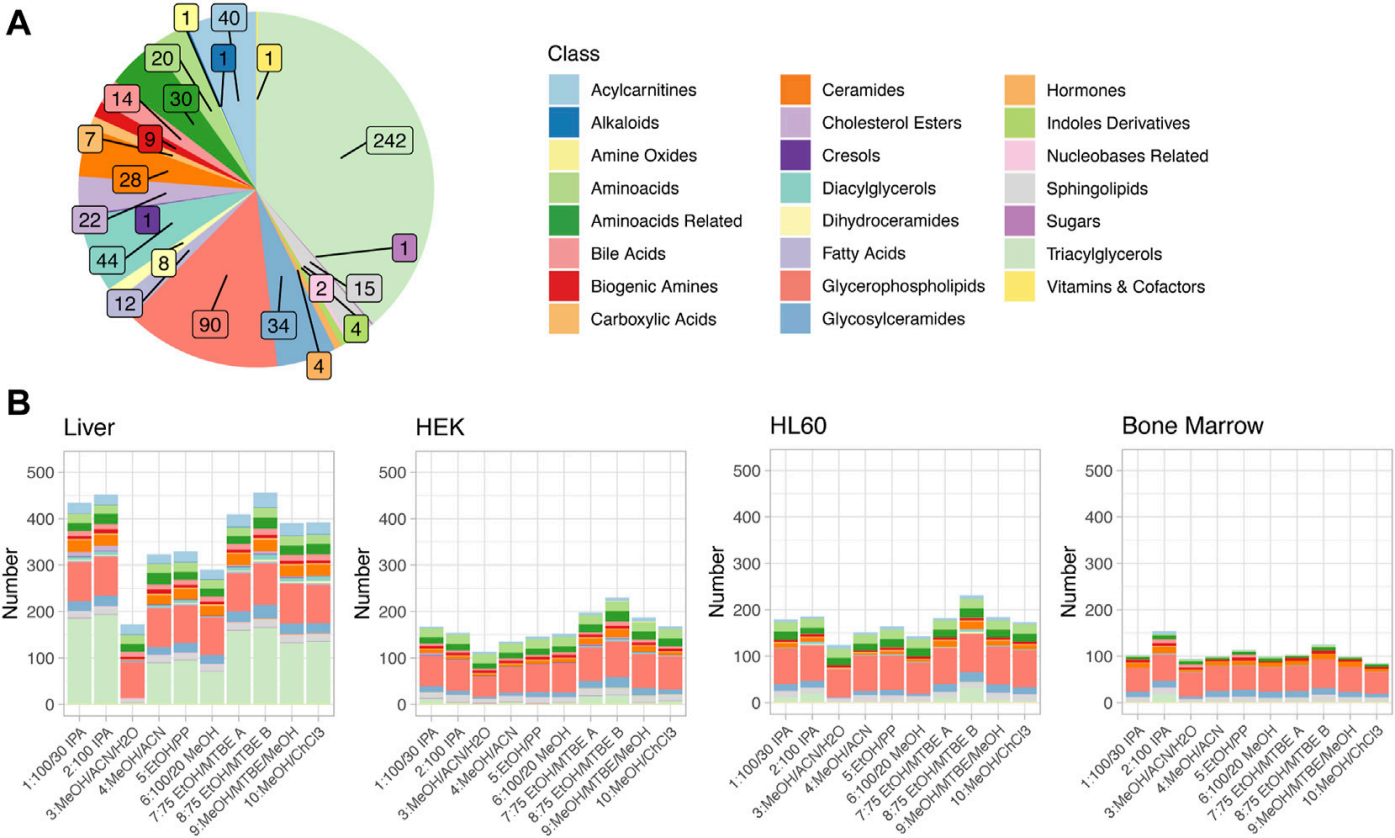
**(A)** Metabolites detectable *via* the Biocrates MxP^®^ Quant 500 kit. Colors encode the different metabolite classes. Numbers of metabolites per class are indicated by the inset. **(B)** Metabolites above the limit of detection (LOD) in the four different sample types and for all extraction protocols. The same color code is used as in **(A)**.

The MxP^®^ Quant 500 kit covers up to 630 metabolites from different chemical classes. These classes with their respective numbers of metabolites are displayed in Figure 2A, highlighting a large fraction of triacylglycerols (242/630 = 38.4%) and glycerophospholipids (90/630 = 14.3%).

**FIGURE 3:**
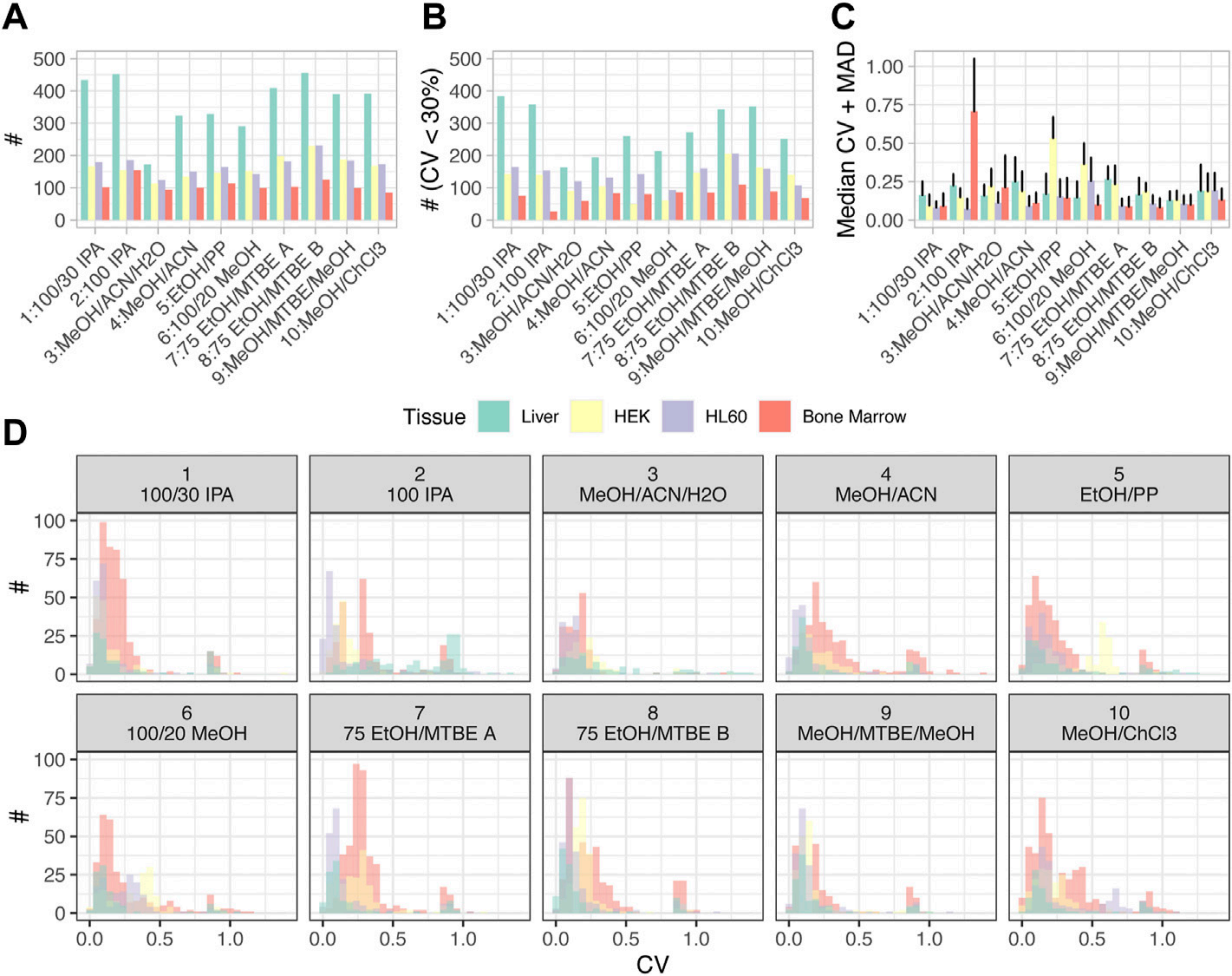
Statistics for each extraction method and tissue type. **(A)** Summary statistics of numbers of metabolites above the LOD. **(B)** Numbers of metabolites with coefficients of variation (CV) below 30%. **(C)** Median and median absolute deviation (MAD) for the CVs. **(D)** Distributions of CVs are displayed as histograms for different tissues and extraction methods (only metabolites above LOD). Color code applies to all items.

Effective quantification of metabolites requires reliable signal strength. The LOD is defined as three times the signal-to-noise of the baseline, calculated by the software MetIDQ for each metabolite. Figure 2B displays the numbers of metabolites above LOD, color-coded by the different metabolite classes, in four different sample types as stacked bar graphs for the different extraction protocols. On average, the highest number of metabolites above LOD was observed for liver tissue (median: 391, range 171–456), and the lowest number was observed in bone marrow (median: 101, range 85–154), while the two investigated cell lines, one adherent (HEK, median: 160.5, range 113–230) and one non-adherent (HL60, median: 176, range 124–231) had very similar and intermediate total numbers of metabolites above LOD. The distribution of metabolites above LOD among the different metabolite classes was similar to the overall distribution of metabolites in the kit (Figures 2A,B), with the important additional observation that the most variable class was triacylglycerols (median ± median absolute deviation (MAD): 3.5 ± 5.2). For the four different sample types, different extraction protocols provided the highest number of metabolites above the LOD: while for liver tissue and HL60, extraction protocols *8: 75 EtOH/MTBE B* and *2: 100 IPA* yielded the highest coverage, protocols *7: 75 EtOH/MTBE A* and *8: 75 EtOH/MTBE B* performed best for the HEK cell line, and *2: 100 IPA* and *8: 75 EtOH/MTBE B* allowed the detection of the highest number of compounds for bone marrow samples. Integrating across the different tissues, protocol *8: 75 EtOH/MTBE B* performed best in terms of the total number of detectable metabolites. Figure 3A also displays the number of metabolites above LOD but aggregated and dodged by extraction protocol. Method *3: MeOH/ACN/H2O* provided the lowest coverage of compounds across all sample types, and all methods containing methanol had comparably lower coverage.

In addition to the total number of detectable analytes, we assessed and compared metabolite concentrations across different protocols. For each metabolite, optimal extraction methods were determined by performing an ANOVA on the measured concentration above the LOD. Extraction methods with either the highest median yield or non-significantly lower concentrations were considered optimal for this compound. When ranking extraction methods using this quality metric, the best extraction methods for each tissue were discovered to be the same ones as those found using numbers of metabolites above LOD; increased heterogeneity was observed in the lower ranking extraction methods. This again confirmed the importance of a suitable extraction protocol (Supplementary Figure S4).

### Repeatability of extraction efficacies between replicates

We next assessed the repeatability of metabolite concentrations obtained *via* different protocols. To this end, we analyzed biological replicates for all sample types and protocols and computed the coefficients of variation (CVs). Figure 3B displays the number of metabolites with CV < 30%, reflecting those metabolites with good repeatability of extraction efficacy. In agreement with the observation that liver tissue provided the highest number of metabolites above LOD in total, liver tissue was also the sample type with the lowest variation in concentrations between replicates. In general, the distribution of the numbers of metabolites with low variation between replicates across the different extraction protocols correlated well with the total numbers of metabolites above LOD (Figures 3A,B). Of note, for some tissues, a large fraction of all metabolites had low variation in concentrations between replicates (i.e., low CVs) in all extraction protocols, for example, for liver tissue, while for other tissues, only small fractions of all metabolites above LOD had low variation between replicates, especially for bone marrow. This was reflected by the overall distribution of the CVs, median, and median absolute deviation (MAD) (Figure 3C), with many combinations of cell type and extraction protocol not only reaching median CVs as low as 20% but also exhibiting marked outliers, like bone marrow in extraction protocol *2: 100 IPA* and *9: MeOH/ACN/H20*, HEK in extraction protocol *5: EtOH/PP* or HL60 in extraction protocol *6: 100/20 MeOH*. Figure 3D shows the distributions of CVs as histograms for the metabolites detected above LOD. It can be observed that for all combinations of sample types and extraction protocols, the distributions of CVs had a long tail toward high CVs, representing few metabolites with low analytical repeatability. Matching the observations described above for Figure 3D, extraction protocol *2: 100 IPA* applied to bone marrow led to a particularly high number of such metabolites. In analogy to Figure 3B; Supplementary Figure S5 displays the histograms of CVs for all metabolites (including those below LOD). As expected, adding metabolites below the LOD threshold populated quantiles of metabolites with high CVs.

### Sum of concentrations as a quality measure

In search of useful and intuitive quality control (QC) metrics, the sum over all metabolite concentrations was calculated to estimate the overall analytical repeatability between biological replicates. The sum of concentrations (SOCs) reflects the variability between replicates and may integrate the variation of multi-step experimental extraction protocols. It also gives an impression of the overall extraction efficiency for different sample types. Supplementary Figure S6 displays this value for three biological replicates for the four sample types and all extraction protocols. In general, depending on protocol and tissue, a good consistency over the biological replicates could be observed. For example, *10: MeOH/ChCl3* shows low variation for the liver and bone marrow (CV: 0.03 and 0.05) but high variation for HEK and HL60 (CV: 0.18 and 0.44). Even though among all extraction protocols *2: 100 IPA* resulted in the best outcome based on the number of metabolites above LOD, the SOC yields similar values for methods 1, 3, 4, 5, 6, and 8 (c.f. Figure 2B). Furthermore, SOC is a metric that is complementary to the number of metabolites above LOD as it aggregates absolute concentrations and thereby makes use of the functionality of the MxP^®^ Quant 500 kit. Due to the different nature of samples, concentrations for HEK, HL60, and bone marrow are given in picomole per 10^6^ cells and in picomole per milligram for liver tissue. Therefore, the SOC for the liver is displayed on a different scale, and the data shown here do not contradict the observation that liver tissue showed the highest number of metabolites above the LOD. However, using published values for hepatocellularity of (65–185) ∙ 10^6^ cells/g (Wilson et al., 2003) or (139 ± 25) **∙** 10^6^ cells/g (Sohlenius-Sternbeck, 2006), the 30 mg of liver tissue correspond to (1.95–5.55) **∙** 10^6^ cells or (4.17 ± 0.75) ∙ 10^6^ cells, respectively, and were in a similar range as the 3 ∙ 10^6^ cells of the other sample types. For HEK, HL60, and bone marrow, there is an overall trend of the SOC from high to low for the sample types. A comparison of the SOC with the number of metabolites above the LOD as a QC metric shows that these two measures did not correlate and that the SOC can provide additional information (e.g., *2: 100 IPA* for HEK and HL60, c. f. Figure 2B; Supplementary Figure S6).

### Metabolite concentrations

**FIGURE 4:**
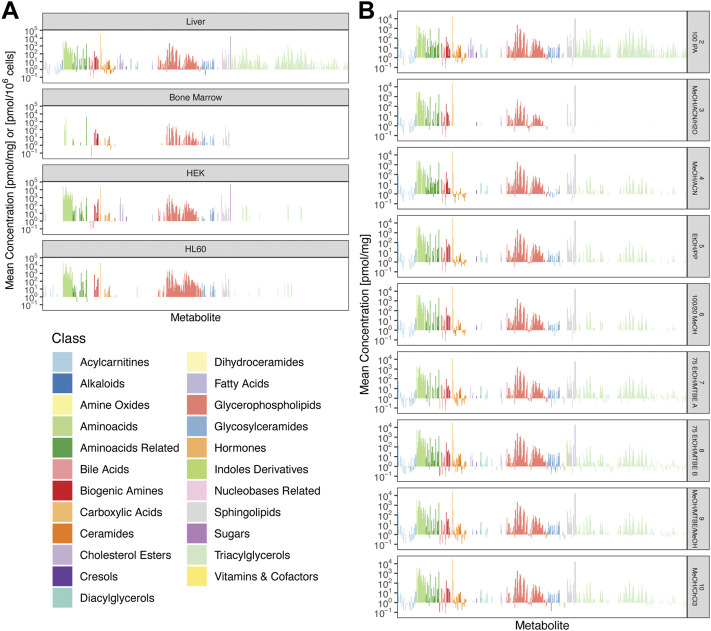
Mean absolute concentrations between replicates. **(A)** Comparison of all four cell types for *8: 75 EtOH/MTBE* **(B)**. Concentrations of bone marrow, HEK, and HL60 samples are plotted as pmol/10^6^ cells and pmol/mg for liver tissue. **(B)** Comparison of all additional extraction protocols for liver tissue. Colors encode metabolite classes as in **(A)**.

Restricting our attention to those metabolites whose concentrations were above LOD and which had acceptable repeatability, we compiled absolute concentrations of these metabolites. Figure 4A shows extraction profiles of these absolute concentrations for all cell types obtained with extraction protocol *8: 75 EtOH/MTBE B*, and Figure 4B shows extraction profiles for all remaining extraction protocols for liver tissue. The complete information for all cell types and all extraction protocols is shown in Supplementary Figure S7. The concentrations in HEK cells showed the broadest range, from 0.015 pmol/10^6^ cells for glycolithocholic acid sulfate (a bile acid) to 68.6 nmol/10^6^ cells for hexose. Even though hexose had the overall highest concentration in liver tissue and HEK cells, in HL60 and bone marrow, the abundance of hexose is below LOD in all extraction methods (Figure 4A; Supplementary Figure S7). In order to visualize varying coverage across metabolite classes using different extraction protocols, we summarized the concentrations of each class of metabolites as box plots (Supplementary Figure S8).

### Variance across extraction profiles is driven by sample type and solvent

**FIGURE 5:**
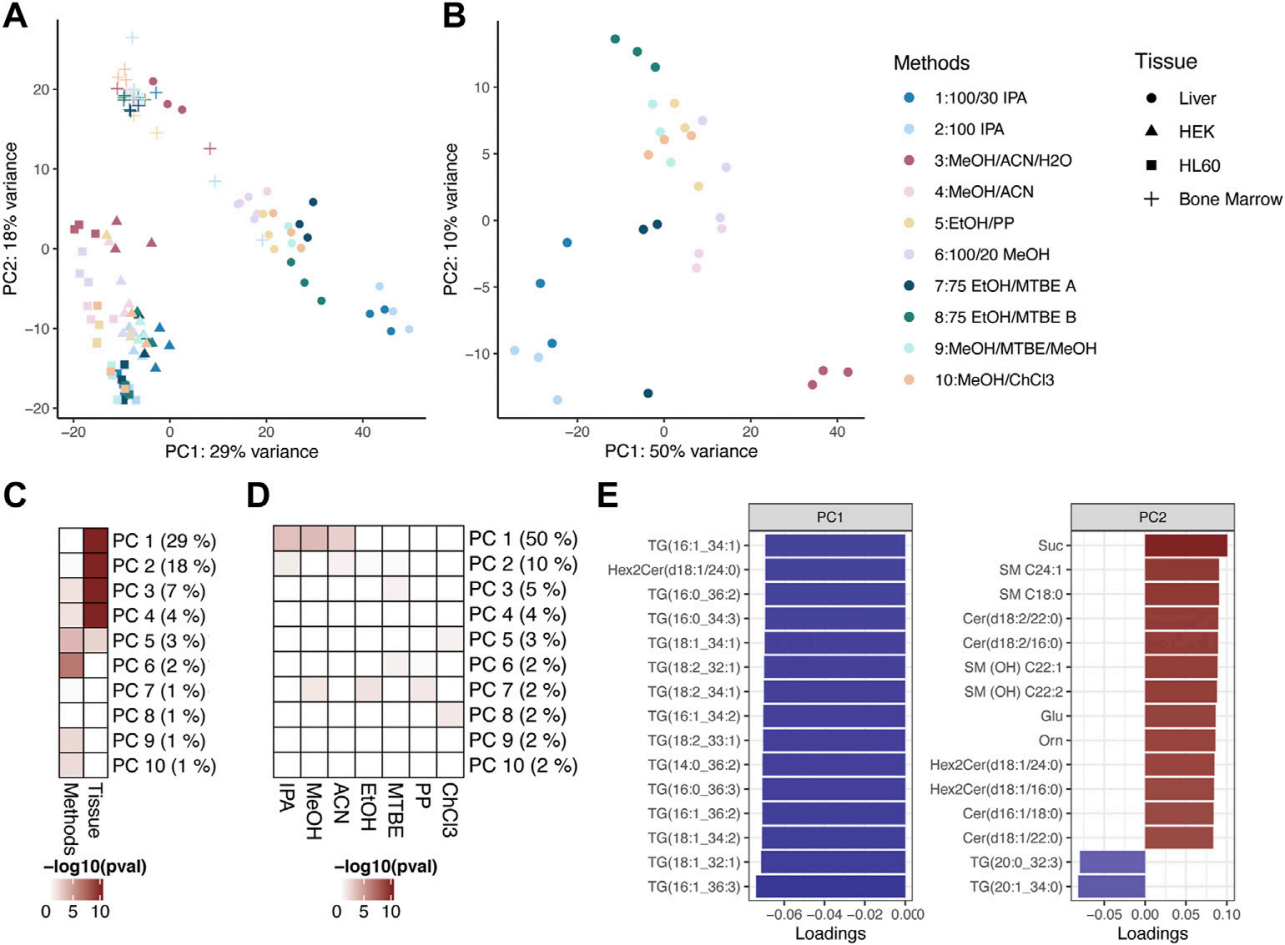
**(A)** PCA of the ten different extraction protocols used across all sample types. **(B)** PCA of the ten different extraction protocols used on liver tissue samples. Colors encode metabolite classes as in **(B)**. **(C)** Heatmap showing association strength between PCs and experimental setting (extraction protocol or tissue), color coding by the *p*-value of the Kruskal-Wallis test. **(D)** Heatmap showing association strength between PCs and solvents used in extraction protocols, color coding by the *p*-value of the Kruskal–Wallis test. **(E)** Barplot of the 15 highest (ranked by absolute value) loadings for PC1 and PC2. The color encodes the signs of the loadings.

An unsupervised principal component analysis (PCA) showed that biological replicates clustered together and that the first five principal components (PCs) had their variance explained mostly by tissue type as the main effect. PC3-6 and PC9-10 reflected the extraction protocol (Figures 5A,C). Focusing only on liver tissue, PCA revealed that similar protocols clustered together and the main variability between the extraction methods is determined by the solvents. PC1, which captures the highest fraction of the total variance, is most strongly associated with IPA, with the next highest association strengths for MeOH and ACN (Figures 5B,D). For PC1, the highest loadings contain multiple triacylglycerides, whereas ceramides and sphingomyelins seem to drive the variability of PC2. This reflects that extraction efficiencies for the various metabolite classes were highly different across the different solvents, depending on the chemical properties of the metabolite classes (Figure 5E; Supplementary Figure S9).

### A resource for interactive exploration

Efficient and repeatable extraction of metabolites is crucial for determining their intracellular abundance. In the previous sections of this manuscript, we have focused on the performance of an extraction method across all metabolites, which might be a compromise when focusing on specific classes of metabolites or distinctive compounds of interest. One method which was very efficient for the extraction of one class of metabolites may provide less optimal results for another one (c.f. Figure 4). For a customized exploration of the dataset, the data presented in Figures 2–4 and in Supplementary Figures S4–S7 were made available as an easy-to-use interactive R/shiny application that allows users to explore and compare the different extraction methods at http://www.metaboextract.shiny.dkfz.de. Tissues, extraction protocols, and classes of metabolites can be (de)selected to focus on the data of interest, and the maximal CV between replicates can be chosen to identify the most suitable method for future analyses. Methods and functions for data processing and conversion were coded in our new R software package MetAlyzer (https://cran.r-project.org/package=MetAlyzer).

## Discussion

While metabolic measurements are well-established for the analysis of body fluids such as blood plasma (Burla et al., 2018), CSF (Blennow and Zetterberg, 2018), or urine (Zapf et al., 2015; Harpole et al., 2016), there is less standardization for extraction of intracellular metabolites and subsequent metabolomic measurements. In a metabolomic study, the pre-analytical phase, that is, the sample collection, handling, and pre-treatment has tremendous effects on the results. While stringent SOPs during sample collection and handling control for this error-prone pre-analytical part (Yin et al., 2015), the adequate choice of an extraction protocol, that is, the pre-treatment of the sample to extract the metabolites of interest on a given analytical platform (Dietmair et al., 2010; Martineau et al., 2011) also influences the range, robustness, and validity of the generated data. Here, we provide a comprehensive analysis of ten different extraction methods for intracellular metabolic measurement in four human sample types: the tissue types liver and bone marrow as well as the cell lines HEK (adherent) and HL60 (non-adherent) using the Biocrates MxP^®^ Quant 500 kit, which allows absolute quantification of up to 630 metabolites *via* LC-MS/MS and FIA-MS/MS measurements.

Assessment of QC metrics (LOD, CV, and SOC) showed that efficiency and repeatability are complementary sources of information. In addition, we used the SOC as a quality metric to assess the global variability between replicates and could show that certain protocols are more prone to technical variability than others. Overall, *2: 100 IPA* and *8: 75 EtOH/MTBE B* showed the best results across the different sample types.

Here, our findings are in line with other extraction comparisons in lipidomics and similarly broad metabolic profiling. Extraction protocols containing isopropanol showed good coverage, low technical variance, and concentrations, making them comparable to MTBE-based extractions (Calderón et al., 2019). Importantly, isopropanol-based monophasic extractions require fewer solvents and are easier to scale up due to their rapid protocol. While MTBE and chloroform extraction (Bligh & Dryer) are comparable in their coverage (see MetaboExtract), MTBE may be seen as a non-toxic alternative that also provides less technical variance (Calderón et al., 2019; Gegner et al., 2022).

For each sample type and metabolite of interest, the optimal method may be different. For liver tissue, *8: 75 EtOH/MTBE B*, *2: 100 IPA*, and *1: 100/30 IPA* resulted in the highest, second highest, and third highest numbers of metabolites above LOD, respectively, with relatively low differences between these three methods. With more than 400 metabolites above LOD for all these methods, almost the full spectrum of the Biocrates MxP^®^ Quant 500 kit can thus actually be exploited for liver tissue. For bone marrow, *2: 100 IPA*, *8: 75 EtOH/MTBE B*, and *5: EtOH/PP* resulted in the highest, second highest, and third highest numbers of metabolites above LOD, respectively, but *2: 100 IPA* performed considerably better than the latter two. With the best methods yielding hardly more than 150 metabolites over LOD, only one-quarter of the metabolites measured by the Biocrates MxP^®^ Quant 500 kit could be found in detectable concentrations. The two different cell lines showed very similar rankings of the different methods, with *8: 75 EtOH/MTBE B* having by far the best yield of metabolites above LOD. With slightly over 200 detectable metabolites *via* this method, the total coverage is slightly better than for bone marrow. Of note, the most variable class of metabolites was triacylglycerols, at least partially reflecting variations in the content of these metabolites across the different tissues. The one method which resulted in the lowest number of detectable compounds across all sample types was *3: MeOH/ACN/H2O*, and all methods containing methanol had comparably lower yields. This could potentially be due to the fact that methanol may not be apolar enough to properly extract the very apolar lipid species.

As expected, our data show that the used solvents influence the profile of extracted metabolites due to the different solvent strength and polarity of the organic solvents themselves or mixtures thereof. To ensure quantitative extraction of very hydrophobic lipid species, apolar solvents such as isopropanol or MTBE—classical solvents used in lipidomics approaches—are required, while in comparison, the influence on extraction efficacy of polar compounds of core carbon and nitrogen metabolism (e.g., amino acids) is minor.

For HEK, HL60, and bone marrow samples, 3 × 10^6^ cells were used as input, whereas the extraction efficiency was significantly higher for liver samples which had 30 mg tissue as the input. This suggests that the extraction efficiency and repeatability can be increased by larger amounts of sample input.

In addition to this comprehensive overview and comparison of the different extraction protocols, we provide a software package for data processing and conversion (R package MetAlyzer) and a free and flexible online resource to allow scientists to explore and subset the data in a tailored manner (R/shiny app MetaboExtract). Using MetaboExtract, the dataset generated here can be visualized and analyzed depending on specific requirements. It enables further in-depth analysis based on a metabolite of interest, a metabolite class, or related to one sample type only. A specific use case could be, for example, a project which aims at measuring amino acids in HL60. Even though method *3: MeOH/ACN/H2O* yields the lowest number of metabolites above LOD in general, for this specific combination of metabolite class and sample type, method *3: MeOH/ACN/H2O* still may be a good choice. In addition to the specific advantages and disadvantages of the different protocols for yielding sufficient numbers of metabolites above LOD, it may be important to also consider the complexity of the protocol and availability of the chemical components required by the different protocols (Figure 1). These two aspects may need to be carefully weighed against each other for every new experimental setting, and we advocate that the interactive shiny app MetaboExtract is a versatile and powerful tool in this process.

In conjunction with the developed software, this work has the potential to inform future studies of the most adequate extraction protocol and optimize the pre-analytical phase in general, that is, also using measurement technologies other than the Biocrates MxP^®^ Quant 500 kit. Due to its flexible nature and design, MetaboExtract may also grow by the inclusion of further datasets, for example, from other tissues or different model organisms and may become a popular and important resource for the planning phase of metabolomic experiments.

## Conclusion

In conclusion, in this work, we provide a comprehensive comparison of different extraction methods for intracellular metabolic measurements by absolute quantification, identify optimal choices of methods for different sample types including primary tissues and cell lines, and provide free software for processing and an online resource for interactive exploration of the data.

## Data Availability

Data can be explored and downloaded using the Shiny app MetaboExtract which is available at http://www.metaboextract.shiny.dkfz.de. The underlying code is also available at MetaboExtract, https://github.com/andresenc/MetaboExtract.
