# Supplementary material for: Comparison of extraction methods for intracellular metabolomics of human tissues
Source: Front Mol Biosci. 2022 Aug 26;9:932261. doi: 10.3389/fmolb.2022.932261 (PMC9461704; doi:10.3389/fmolb.2022.932261)
Supplement: Supplementary file 1 [file Presentation1.PDF]

Supplementary Items

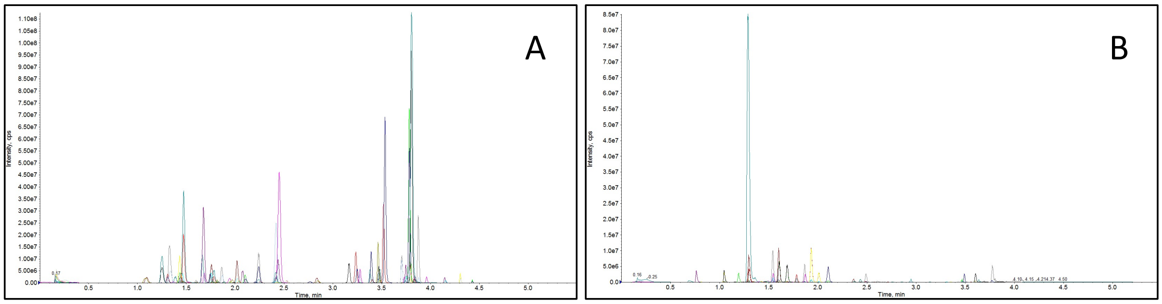

Supplementary Figure 1. Chromatogram of quality control (QC2) sample obtained by UPLC-MS/MS. (A) Analysis performed in positive ionization mode (125 MRMs for compounds of interest and internal standards). (B) Analysis performed in negative ionization mode (69 MRMs for compounds of interest and internal standards).

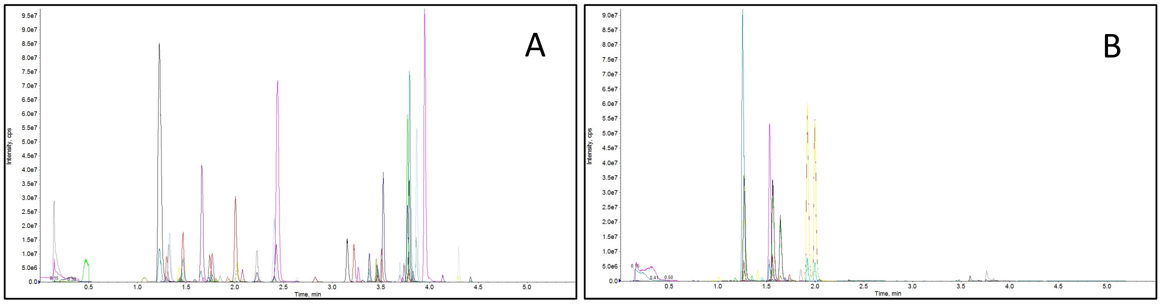

Supplementary Figure 2. Example chromatogram of human liver sample obtained by UPLC-MS/MS. (A) Analysis performed in positive ionization mode. (B) Analysis performed in negative ionization mode.

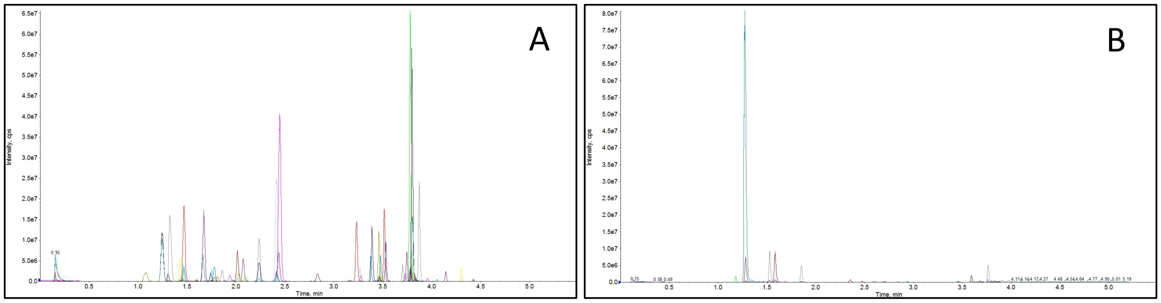

Supplementary Figure 3. Example chromatogram of HEK cells sample obtained by UPLC-MS/MS. (A) Analysis performed in positive ionization mode. (B) Analysis performed in negative ionization mode.

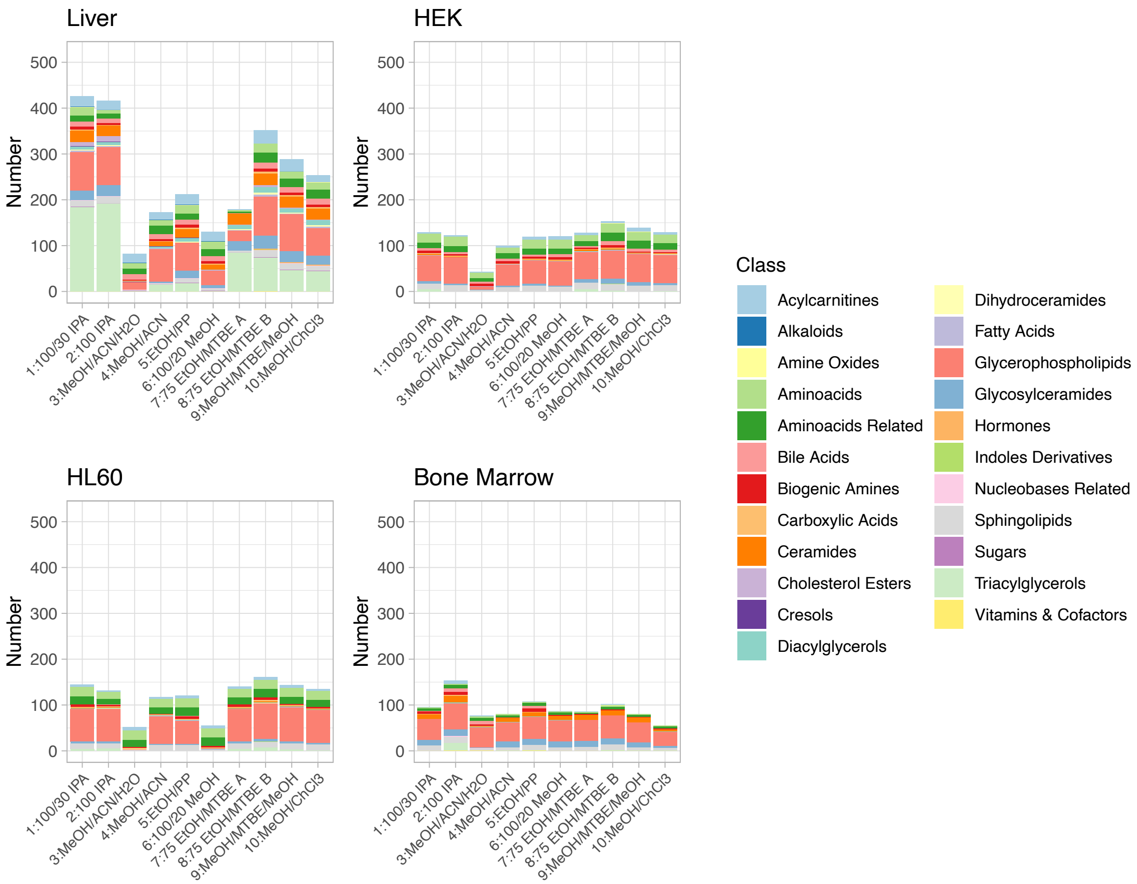

Supplementary Figure 4. Metabolites above limit of detection (LOD) with highest concentration yield among extraction protocols in the four different sample types calculated by ANOVA. Colors encode the different metabolite classes.

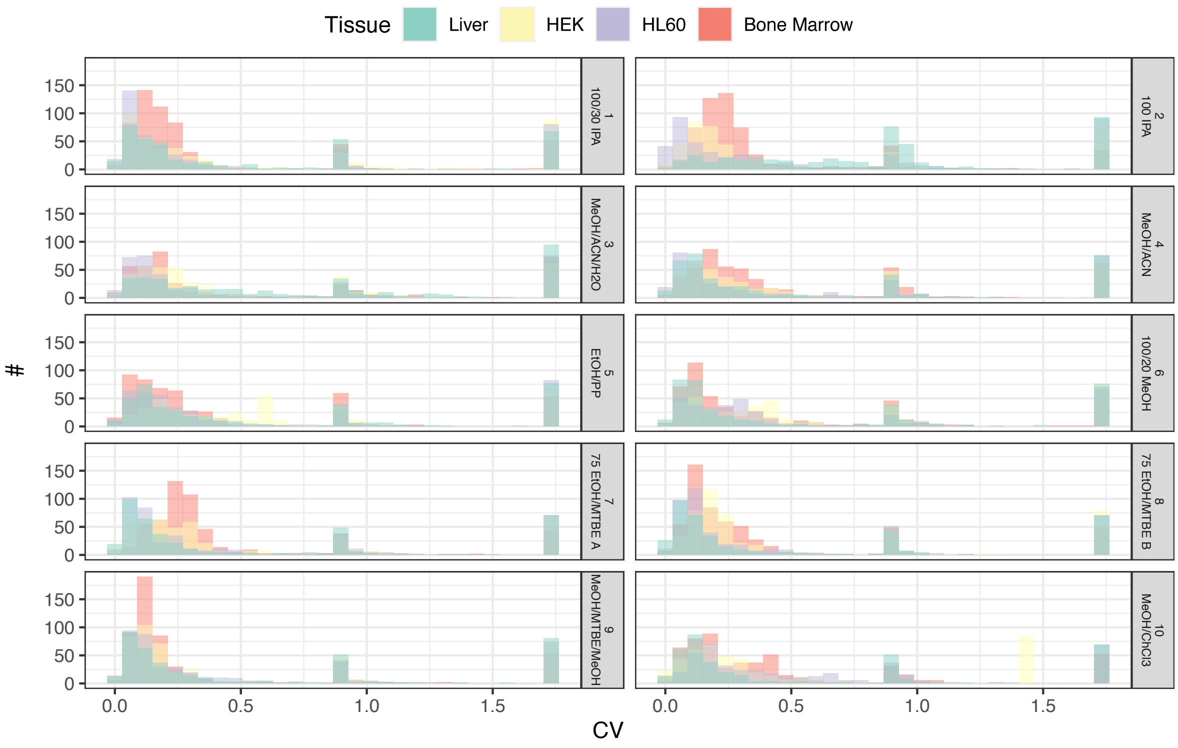

Supplementary Figure 5. Distributions of CVs between replicates across extraction protocols and sample types.

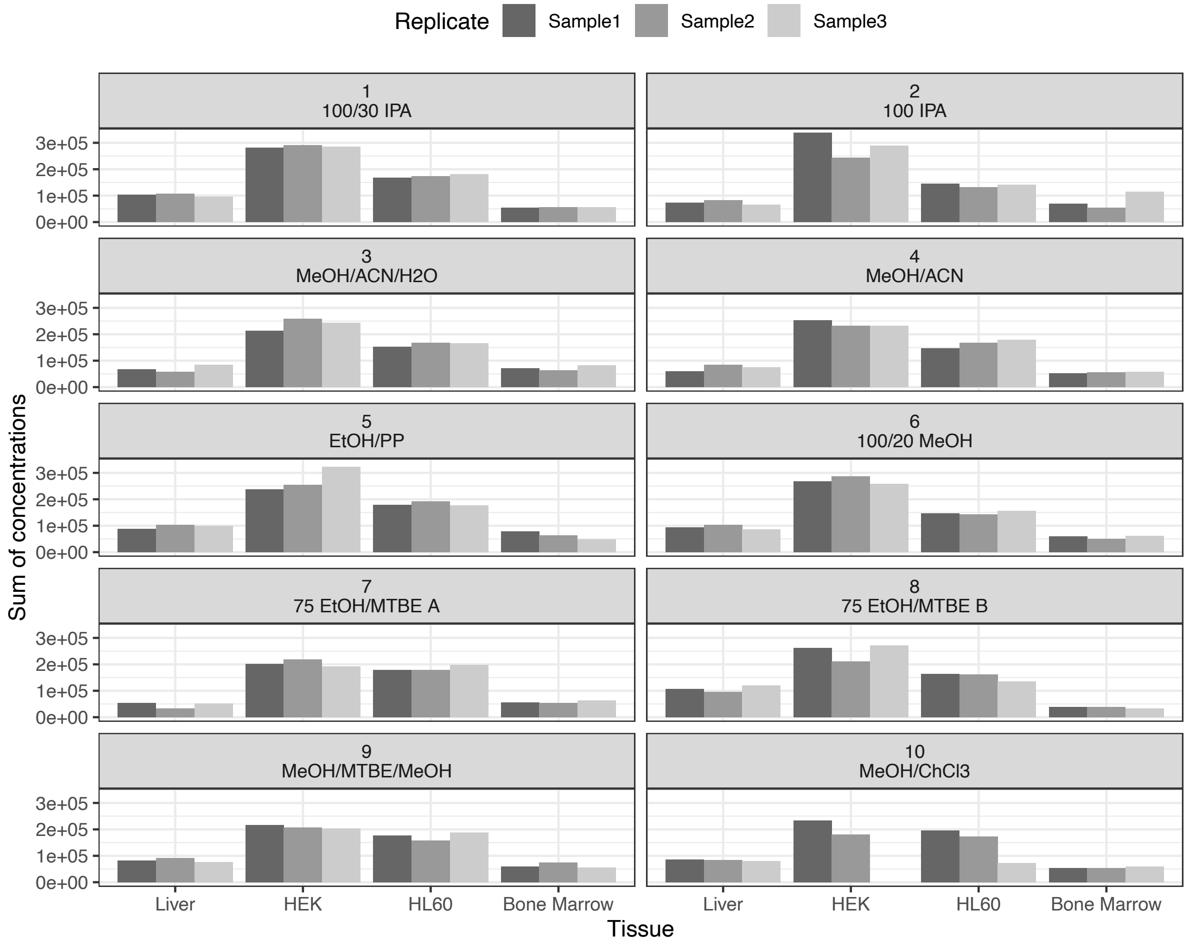

Supplementary Figure 6. Sums of concentrations between replicates across extraction protocols and sample types. Of note, one replicate of HEK was removed because of very low concentrations.

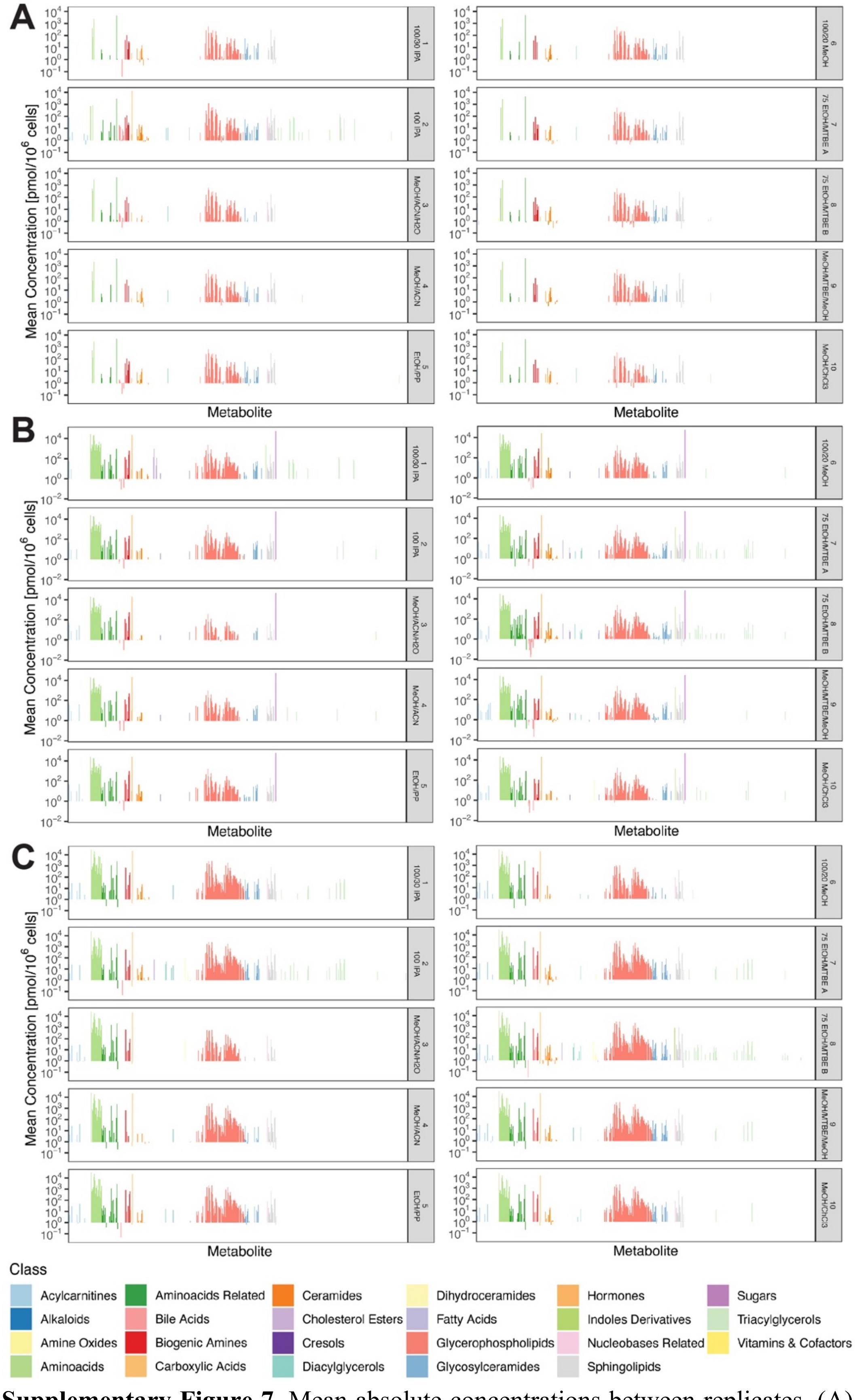

Supplementary Figure 7. Mean absolute concentrations between replicates. (A) Bone marrow, (B) HEK and (C) HL60.

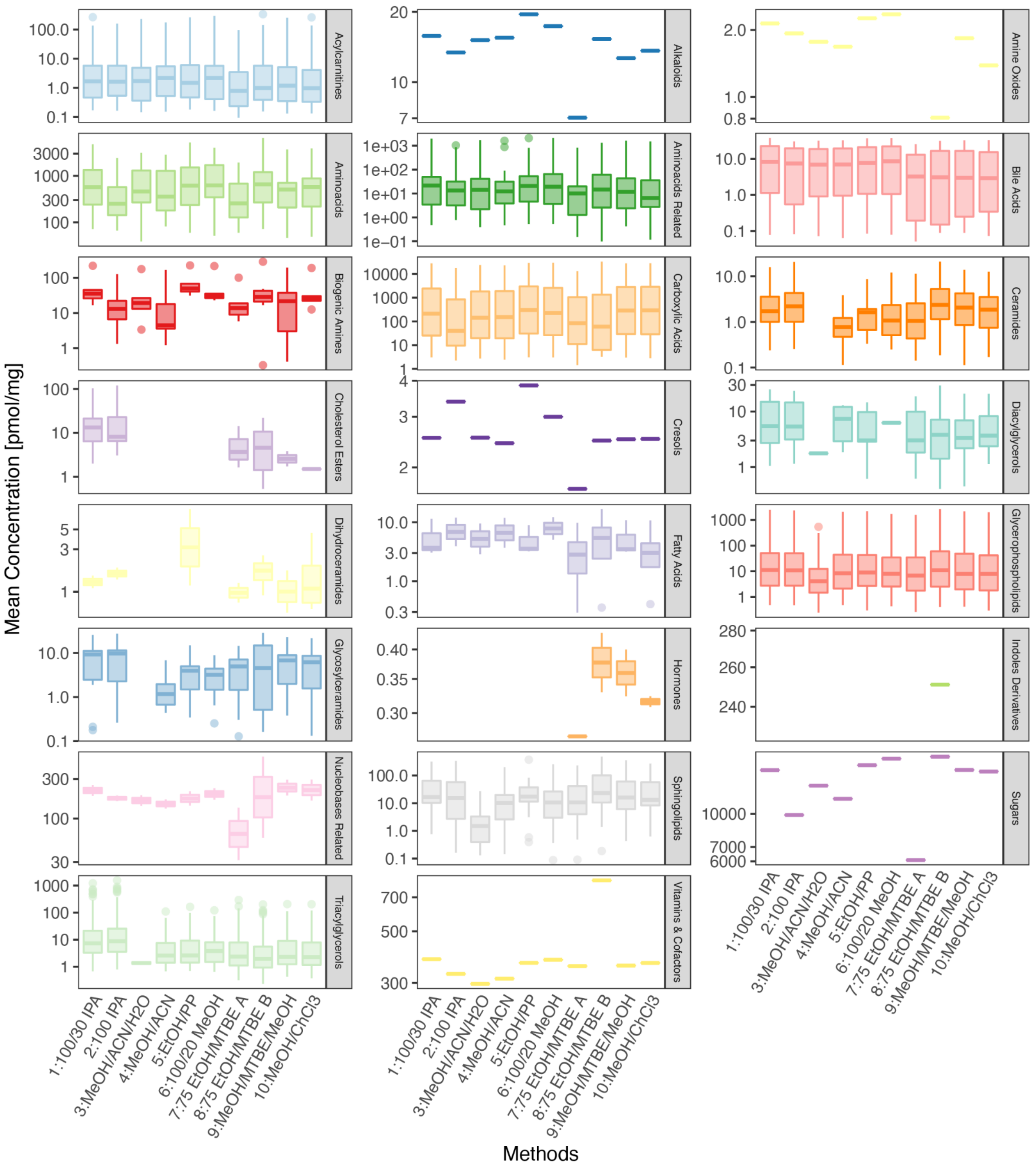

Supplementary Figure 8. Varying coverage across metabolite classes using different extraction protocols in liver tissue. Concentrations of each class of metabolites are summarized as box plots.

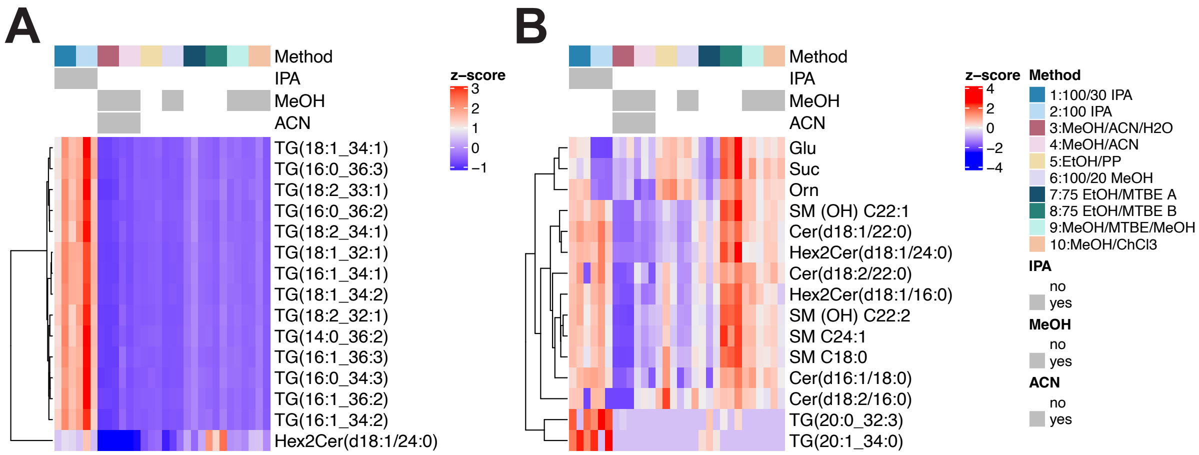

Supplementary Figure 9. Concentrations of the 15 highest (ranked by absolute value) loadings (metabolites) for (A) PC1 and (B) PC2. Methods and selected solvents are encoded in (B).
